# 亲水作用液相色谱分离纯化南极磷虾油中的磷脂

**DOI:** 10.3724/SP.J.1123.2026.04010

**Published:** 2026-08-08

**Authors:** Jian PAN, Lin HONG, Yan ZHANG

**Affiliations:** 1. 大连凌水湾实验室，辽宁 大连 116000; 1. Dalian Lingshui Bay Laboratory，Dalian 116000，China; 2. 大连市检验检测认证技术服务中心，辽宁 大连 116000; 2. Dalian Center for Certification and Food and Drug Control，Dalian 116000，China; 3. 海正药业（杭州）有限公司，浙江 杭州 311404; 3. Hisun Pharmaceutical （Hangzhou） Co.，Ltd.，Hangzhou 311404，China

**Keywords:** 海洋磷脂, 南极磷虾油, 亲水作用色谱, 二醇基固定相, 制备高效液相色谱, marine phospholipids, Antarctic krill oil, hydrophilic interaction chromatography （HILIC）, diol stationary phase, preparative high performance liquid chromatography （Prep-HPLC）

## Abstract

南极磷虾油富含磷脂，是海洋磷脂的重要来源，且此类磷脂与二十碳五烯酸（EPA）、二十碳六烯酸（DHA）结合产生磷脂型EPA、DHA，更易被人体吸收利用，发挥良好的生物活性功能。本研究建立了从南极磷虾油中纯化制备高含量磷脂的亲水作用色谱方法。该方法以二醇基（diol）键合硅胶为固定相，乙醇和水为流动相，采用3个台阶梯度洗脱的方式对南极磷虾油进行分离纯化。实验结果表明终产品的产率为49.15%。经钼蓝比色法（GB/T 5537-2008）测定，产品中磷脂含量由57.88%提升至95.42%，磷脂回收率为81%；经外标定量法（GB 5009.168-2016）测定，产品中EPA含量为18.5%，DHA含量为7.18%。该方法可高效获得药用级纯度的磷脂，具备环境友好性与工艺放大潜力。本文提供了以南极磷虾油为代表的海洋磷脂的绿色纯化制备新路径，为相关产品质量标准的提高奠定了关键技术基础。

## Abstract (en)

Antarctic krill has the largest biomass of any wild animal species on Earth. Krill oil extracted from Antarctic krill is rich in phospholipids， and is a major natural source of marine phospholipids. In marine phospholipids， eicosapentaenoic acid （EPA） and docosahexaenoic acid （DHA） are esterified at the *sn*-2 position of glycerol. This unique structural configuration endows them with the dual physiological functions of both phospholipids and *Ω*-3 polyunsaturated fatty acids. Compared with terrestrial phospholipids， marine phospholipids exhibit superior bioactivity in several key aspects， including neurotrophic support， anti-atherosclerotic effects， and lipid metabolism regulation， thereby conferring significant application value in pharmaceuticals， health foods， and functional foods. However， pharmaceutical excipient applications require phospholipid purity above 90%， a standard also required for high-end pharmaceutical formulations and active pharmaceutical ingredients. Currently， commercial krill oil products vary significantly in quality. Their total phospholipid content ranges from only 30% to 60%. Therefore， purifying phospholipids from Antarctic krill is essential. This process holds practical value for improving the quality standards of marine phospholipids. Hydrophilic interaction liquid chromatography （HILIC） separates polar compounds through multiple mechanisms， relying on polar interactions between the hydrophilic headgroups of phospholipids and the stationary phase， as well as partitioning within the water-rich layer on the stationary phase surface. In this study， a HILIC-based method was developed for the purification of phospholipids from Antarctic krill oil. Industrial-scale preparative chromatography requires stable stationary phases. Three HILIC-compatible stationary phases were systematically evaluated. These were unmodified bare silica gel， Diol （3% carbon）， and Diol-H （7% carbon）. Diol showed favorable retention and resolution. Therefore， Diol was selected as the optimal stationary phase. The superior performance of the Diol phase compared to Diol-H suggests that a moderate carbon content is crucial. Excessive hydrophobicity in Diol-H likely interfered with the specific hydrogen bonding interactions required for separating polar lipid headgroups. Ethanol and water were chosen as the mobile phases， because these solvents have low toxicity. Ethanol-water proportions were investigated to develop a three-step elution program： washing with 100% ethanol， elution with 95% ethanol， and column re-equilibration with 80% ethanol. Based on chromatographic plate and rate theories， a Diol column （250 mm×4.6 mm） was used. Isocratic elution with 95% ethanol was applied. The relationship between flow rate， theoretical plate number， and peak width was evaluated. An optimal flow rate was selected. Peak broadening under high sample loading was examined. This determined the maximum loading capacity. It was optimized at 8% of the stationary phase mass. Separation remained efficient at this level. Resolution did not decrease significantly. The final purification protocol involved washing with 2 column volumes of 100% ethanol， eluting the target phospholipid fraction with 5.5 column volumes of 95% ethanol， and re-equilibrating the column with 2.5 column volumes of 80% ethanol. Finally， scale-up separation was performed using a DAC50 column. The crude oil contained 57.88% phospholipids. Four fractions were collected based on online chromatograms. Each fraction was concentrated， dried， and then analyzed. The recovery of the purified product was 49.15%. Purity was measured using the molybdenum blue colorimetric method （GB/T 5537-2008）. Purity reached 95.42%. The overall phospholipid recovery was 81%. EPA and DHA were quantified via external standard method （GB 5009.168-2016）. EPA and DHA contents were 18.5% and 7.18%， respectively. This method efficiently prepares pharmaceutical-grade phospholipids. The process is environmentally friendly and easily scalable for industrial manufacturing. This approach supports the purification of marine phospholipids from Antarctic krill oil and the high-purity concentrate serves as a reference material. It supports clinical research on krill-derived lipids and promotes applications in drug delivery and nutraceuticals. This work improves quality standards for marine lipid products and contributes to the sustainable use of Antarctic krill resources. Preparative liquid chromatography is a mature technology widely used for separating complex samples like drugs and natural products. This study investigated HILIC for purifying phospholipids from Antarctic krill oil. The research provides a technical basis for selecting preparation methods.

在全球所有野生动物中，南极磷虾拥有最大的生物量^［1］^，其提取物南极磷虾油中富含磷脂，是海洋磷脂的重要来源^［2］^。海洋磷脂的甘油结构骨架*sn*-2位置结合有二十碳五烯酸（eicosapentaenoic acid，EPA）、二十碳六烯酸（docosahexaenoic acid，DHA），兼具磷脂与EPA、DHA的双重生理功能。与陆地磷脂相比，海洋磷脂在神经营养、动脉粥样硬化预防、改善脂肪代谢等方面表现更优^［3-7］^，在医药、保健食品及功能食品等领域具有应用价值。在药用辅料（如脂质体、mRNA疫苗载体）、高端制剂、原料药（如注射用磷脂）方面的应用需要含量大于90%的高纯度磷脂^［8-10］^，但目前市售磷虾油产品的质量参差不齐，核心活性成分总磷脂含量仅为30%~60%^［11，12］^。因此，南极磷虾中磷脂的纯化制备对海洋磷脂的质量标准提升具有重要的实际应用价值。

液-液萃取法是富集磷脂的传统方法^［13］^，但回收率偏低，得到的磷脂含量低于90%^［14］^。近年来，有研究对该技术进行改进，如李志刚等^［15］^采用Na_2_CO_3_盐溶液、乙醇、异辛烷与南极磷虾油提取物混合，形成4个液相层进行分离，但该方法易形成乳化层导致目标物损失。此外，朴镇锡等^［16］^采用超临界流体萃取技术提取海洋磷脂；李明等^［17］^采用硅胶柱层析法制备高纯度磷虾磷脂，由于使用了毒性试剂二氯甲烷和甲醇，限制了该方法在食品工业中的应用。当前，制备型高效液相色谱技术凭借其高分离效能已成为发酵药物和天然产物等复杂样品纯化的核心技术之一。本文根据磷虾油中各类物质的理化性质差异，针对磷脂的纯化需求，发展了亲水作用色谱法（hydrophilic interaction chromatography，HILIC）。根据HILIC的分离机理，磷脂的亲水头基可与固定相的极性官能团产生多种作用，结合在富水层中的分配效应，从而实现保留和分离^［18］^。本文开展了基于制备型亲水作用色谱法对南极磷虾油中磷脂的纯化研究，从HILIC的关键因素出发，考察填料类型、流动相比例、洗脱流速、载样量的影响，最终得到高含量磷脂产品。本研究发展的HILIC方法具备环境友好性与工艺放大潜力，可为海洋磷脂的液相色谱制备工艺提供参考。

## 1 实验部分

### 1.1 仪器、试剂与材料

制备液相色谱仪，包括LC-3000制备型液相色谱双泵、紫外检测器、DAC50动态轴向压缩柱（北京创新通恒科技有限公司）；Alliance高效液相色谱仪，包括四元梯度泵、自动进样器、柱恒温系统、2489双波长检测器和Empower工作站（美国Waters公司）；RV 3 V Starter Solution真空旋转蒸发仪（德国IKA公司）；600E气相色谱仪，配FID检测器（美国Waters公司）；JIDI-20D离心机（广州吉迪仪器公司）；UV1900型双光束扫描型紫外可见分光光度计（中福环保仪器公司）；BFC马弗炉（科幂仪器公司）；IQ7000超纯水净化系统（Sigma-Aldrich公司）。

色谱纯正庚烷，以及分析纯氢氧化钠、三氟化硼、甲醇、无水硫酸钠、氯化钠、硫酸、钼酸钠、硫酸联氨、磷酸二氢钾、盐酸、氢氧化钾、氧化锌、乙醇均购于国药集团化学试剂有限公司。Diol填料（粒径30 μm）购于浙江华谱新创科技有限公司。南极磷虾油（总磷脂含量58%）购于山东海龙元生物科技有限公司。二十碳五烯酸甲酯（99%）、二十二碳六烯酸甲酯（99%）购于上海阿拉丁生化科技股份有限公司。

### 1.2 样品溶液的配制

称取南极磷虾油100 mg，溶于10 mL乙醇中，用0.45 μm微孔滤膜过滤，得到10 mg/mL样品溶液用于色谱条件考察。称取南极磷虾油25 g，溶于240 mL乙醇中，用0.45 μm微孔滤膜过滤，得到104 mg/mL样品溶液用于制备色谱分离纯化实验。

### 1.3 色谱条件

#### 1.3.1 分析色谱条件

色谱柱：Diol（表面碳含量3%的二醇基填料）色谱柱（250 mm×4.6 mm，10 μm，浙江华谱新创科技有限公司）；柱温为30 ℃；流动相：乙醇（A）和纯水（B）；洗脱程序：0～30 min，100%A~50%A；流速：1 mL/min；检测波长：210 nm；10 mg/mL样品溶液进样量：20 μL。

#### 1.3.2 制备色谱条件

将300 g Diol填料（30 μm）装填到DAC50动态轴向压缩柱（250 mm×50 mm）中。进样泵进样，样品质量浓度100 mg/mL，进样体积240 mL；流动相：乙醇（A）和纯水（B），制备程序：0~9 min，进样；9~29 min，100%A；29~84 min，95%A；84~110 min，80%A；流速30 mL/min；检测波长：210 nm。收集13~29 min的100%乙醇淋洗液（Washing solution）、29~55 min的95%乙醇洗脱液（Eluent Ⅰ）、55~95 min的95%乙醇洗脱液（Eluent Ⅱ）、95~110 min的80%乙醇洗脱液（Eluent Ⅲ）。将4段馏分分别用真空旋转蒸发仪进行减压浓缩，所得干燥样品检测总磷脂、EPA、DHA含量。

### 1.4 含量检测条件

#### 1.4.1 总磷脂含量检测

参照GB/T 5537-2008《粮油检验磷脂含量的测定》中的第一法（钼蓝比色法）进行供试样品处理和总磷脂含量检测。分别称取南极磷虾油原样、Washing solution样品、Eluent Ⅰ~Ⅲ样品0.3 g，分别置于陶瓷坩埚中，加入氧化锌0.2 g。先在电炉上小火加热，炭化至无烟。将坩埚送至565 ℃马弗炉中灼烧3~5 h，取出坩埚冷却后，用10 mL盐酸溶液溶解灰分并加热保持微沸5 min，待溶液温度恢复至室温后，转移至100 mL容量瓶中，用热水洗涤坩埚3次，一并转移至容量瓶中。用50%氢氧化钾溶液中和至出现浑浊，再加入盐酸溶液使氧化锌沉淀完全溶解，加水定容至刻度，备用。采用分光光度法，记录650 nm处吸光值，测出总磷脂含量。

#### 1.4.2 EPA、DHA含量检测

依据GB 5009.168-2016《食品安全国家标准 食品中脂肪酸的测定》第二法（外标法）处理供试样品和EPA、DHA含量检测。分别准确称取南极磷虾油原样、Washing solution样品、EluentⅠ~Ⅲ样品0.2 g，置于150 mL具塞烧瓶中，在烧瓶中加入8.0 mL 2%氢氧化钠甲醇溶液，涡旋混匀，80 ℃水浴回流，直至油滴消失；从回流冷凝器上端加入7 mL 15%三氟化硼甲醇溶液，于80 ℃水浴中继续回流2 min。用少量水冲洗回流冷凝器。停止加热，迅速冷却至室温。准确加入10 mL正庚烷，振摇2 min，再加入饱和氯化钠水溶液，静置分层。吸取上层正庚烷提取溶液5 mL，至25 mL试管中，加入4 g无水硫酸钠，振摇1 min，静置5 min，吸取上层溶液到进样瓶中待测。

采用强极性毛细管柱气相色谱分离，氢火焰离子化检测器检测，外标法定量测定EPA、DHA含量。

## 2 结果与讨论

### 2.1 亲水作用色谱固定相的筛选

常见的亲水作用色谱固定相包括硅胶、氨基、酰胺、二醇基、两性离子和氰基。氰基柱的亲水性较弱，保留能力有限；氨基柱稳定性较差，键合的氨基易脱落；两性离子型填料表面同时含正负电荷，更适合离子型化合物的分离。有文献报道采用正相模式硅胶柱层析法制备高纯度磷虾磷脂^［17］^，而本实验着重考察Silica（250 mm×4.6 mm，10 μm）、Diol（250 mm×4.6 mm，10 μm，表面碳含量3%）、Diol-H（250 mm×4.6 mm，10 μm，表面碳含量7%）色谱柱在HILIC模式下的分离表现，并重点对比表面碳含量3%和7%的二醇基柱在相同洗脱条件下对磷虾油的分离情况（见图1）。

**图1:**
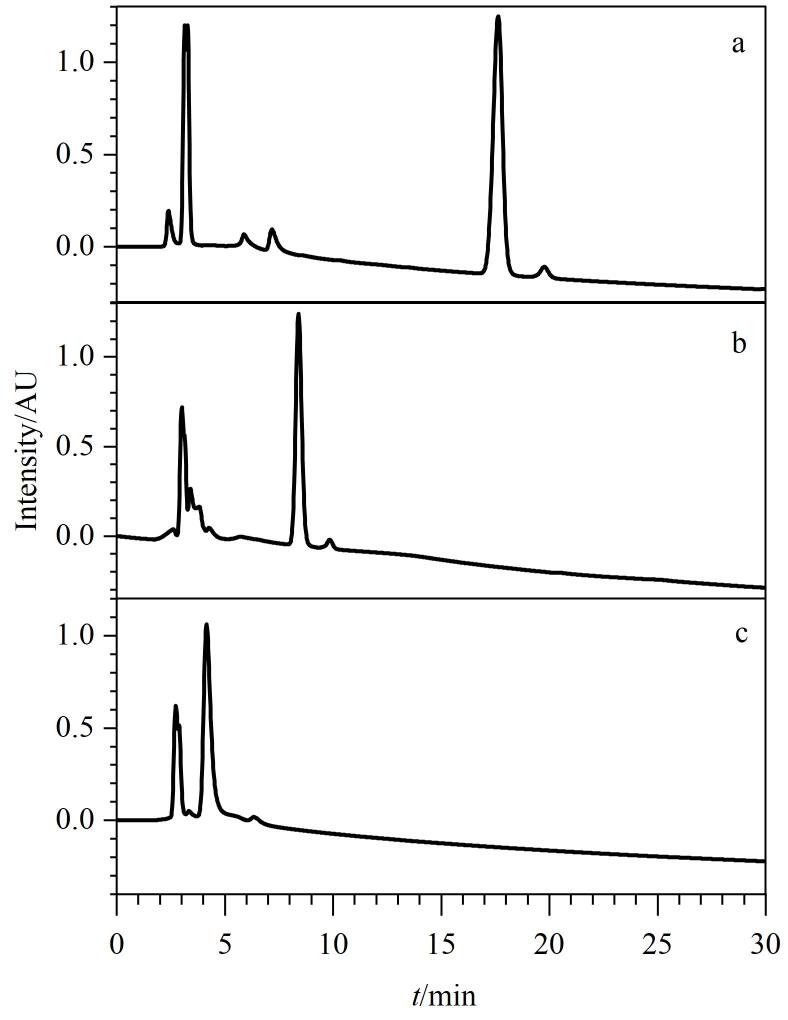
色谱柱类型对磷虾油分离的影响

HILIC的核心保留机理是在高比例有机流动相中，固定相表面会吸附水分子形成稳定的富水层，极性溶质在富水层和高比例有机相间分配，保留强度与溶质的亲水性正相关；同时，溶质的极性官能团与固定相上的极性修饰基团间发生直接相互作用，包括氢键、偶极-偶极、静电吸引/排斥作用等。最终，溶质按照极性由弱到强的顺序被洗脱。南极磷虾油是包含极性脂质（如磷脂，尤其是磷脂酰胆碱）、中性脂质（甘油三酯、胆固醇酯）、游离脂肪酸及类胡萝卜素（如虾青素）的复杂天然油脂，相比于中性脂质，磷脂具有头部亲水端，使其在HILIC色谱柱上的保留时间更长，由此可确定图1中保留时间较长的色谱峰是磷脂。Silica柱对磷脂的保留最长，需要用含水比例较高的流动相才能将所有物质完全洗脱（图1a）。硅胶易吸水，受水分影响，重复性较差，不利于长期稳定使用；同时磷脂在含水较高的流动相中溶解性较差，易析出堵塞色谱柱，因此Silica柱不予考虑；图1b中Diol柱对磷脂与中性脂质分离效果较好，由于保留较硅胶柱弱，洗脱磷脂时流动相中乙醇的比例相对较高，可以较好地溶解磷脂，避免样品析出；Diol-H柱表面键合的二醇基更多，对磷脂的保留最弱，导致磷脂与中性脂质的分离度较小（图1c）。综合对比，选择Diol柱作为亲水作用液相色谱柱进行分离条件的优化，并确定Diol填料作为制备柱的固定相。

### 2.2 制备色谱条件的优化

#### 2.2.1 洗脱程序的优化

制备色谱条件与分析色谱条件的优化侧重点有所差异，除了达到分离效果之外，还需兼顾操作简便性、流动相用量的经济性、单位时间纯化效率的合理性等。基于磷虾油样品中成分的极性跨度较大的特点，设计多个台阶梯度的洗脱条件，分别实现去除中性脂质、分离与磷脂保留时间相近的杂质，以及冲洗再生色谱柱的目的。根据图1b中磷脂的保留时间计算出对应流动相中乙醇体积分数为90%，据此，采用Diol色谱柱（250 mm×4.6 mm，10 μm），考察等度洗脱条件下乙醇比例（80%、90%、95%、100%）对磷虾油分离的影响（图2）。其余条件同1.3.1节。

**图2:**
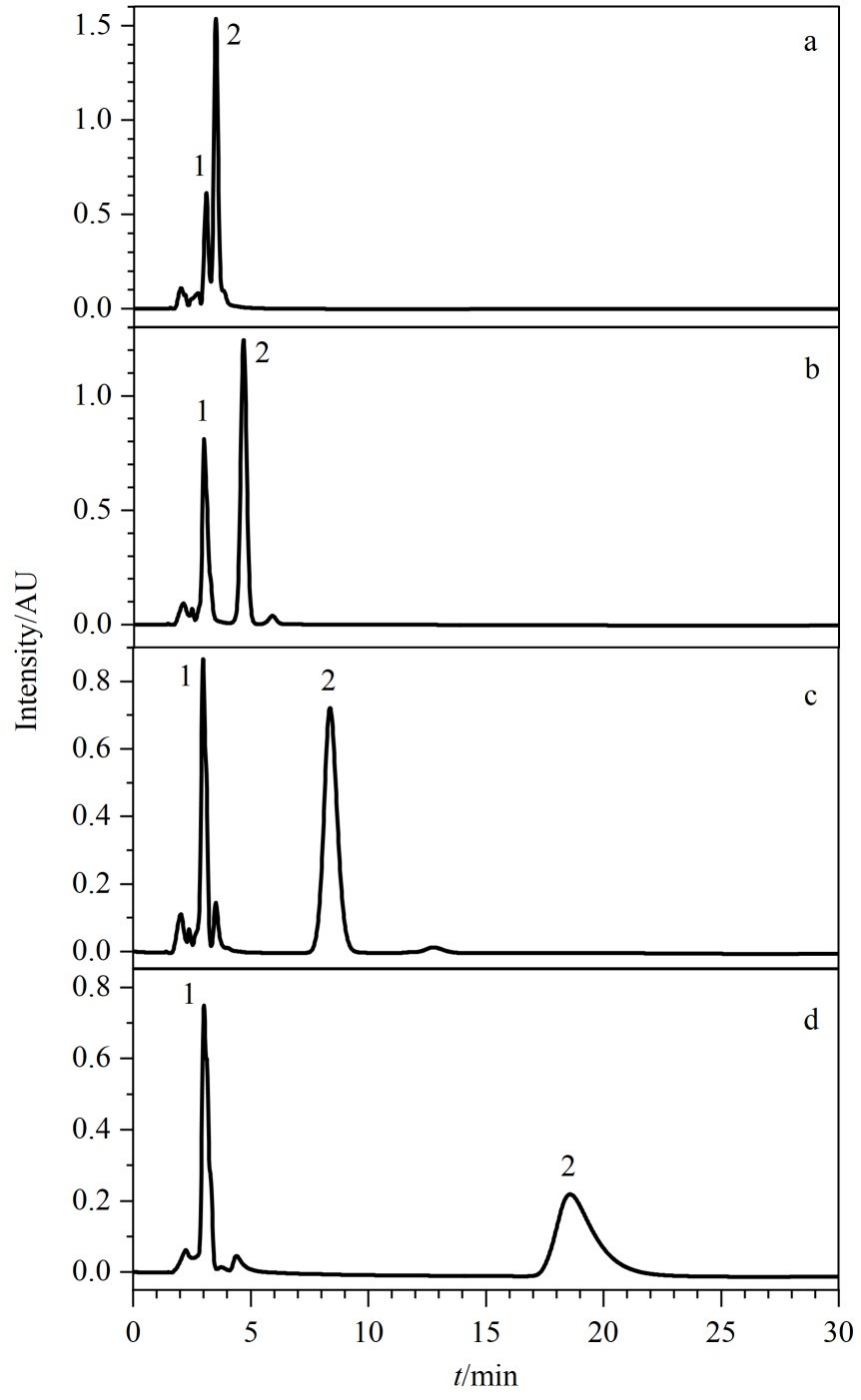
流动相中乙醇比例对磷虾油分离的影响

图2a和图2b表明，在80%乙醇和90%乙醇等度洗脱条件下，流动相洗脱能力较强，特别是80%乙醇，既保证了较高的有机相比例对磷虾油样品中各类物质的溶解性，又可以将各类物质快速洗脱，适合作为Diol色谱柱的冲洗再生流动相。95%乙醇等度洗脱条件下（图2c），主要的磷脂峰（峰2）保留时间为7 min，峰形及与前峰（峰1）的分离度均较好，可以满足分离要求。图2d表明，如果采用100%乙醇流动相洗脱，保留时间18.5 min的磷脂峰与前峰分离度最大，但此时磷脂峰较宽，拖尾也比较严重，洗脱效率不如95%乙醇。综合对比，确定设计3个台阶梯度洗脱作为分离纯化流动相条件，即首先采用100%乙醇洗脱图2d中保留时间小于5 min的色谱峰（多为中性脂质），再换用95%乙醇分离洗脱磷脂，最后使用80%乙醇冲洗再生色谱柱。

#### 2.2.2 制备流速的考察

采用Diol色谱柱（250 mm×4.6 mm，10 μm）进行实验，考察流速对色谱分离的影响，以95%乙醇水溶液为流动相等度洗脱，流速为1.0、0.5、0.3、0.2 mL/min时，以南极磷虾油中磷脂峰计算理论塔板数与峰宽（见表1）。理论塔板数和峰宽均随流速的降低而增加；流速0.2 mL/min条件下，理论塔板数最高，对应峰宽也最大，与流速0.3 mL/min条件相比，其理论塔板数增加1.19倍时，峰宽增加了1.59倍，对于制备色谱而言，峰宽的过度增加是不利因素。综合考虑理论塔板数与峰宽2个参数，本实验选择0.3 mL/min作为磷虾油制备的流速条件。

**表1:** 不同流速下磷脂主峰的保留时间、理论塔板数和峰宽

Flow rate/（mL/min）	Retention time/min	Theoretical plate number/（plate/m）	Peak width/s
1.0	8.37	3902.36	121
0.5	16.65	6107.04	204
0.3	27.12	8029.64	314
0.2	43.92	9545.40	500

#### 2.2.3 载样量的考察

样品制备与分析最大的区别在于载样量不同，载样量定义为上样的样品质量与填料质量的百分比。为了提高制备的效率、降低生产成本，制备液相色谱通常是在过载情况下开展的，其载样量的优化需要结合产品纯度和回收率进行平衡考虑。本实验采用Diol填料（30 μm）装填DAC50动态轴向压缩柱（250 mm×50 mm），样品含量为100 mg/mL，考察了进样体积分别为75、150、240 mL，对应载样量分别为2.5%、5%和8%时的分离情况（图3）。随着载样量的增加，色谱峰随之展宽。图3a为载样量2.5%时的分离情况，中性脂质被100%乙醇在29 min前淋洗下来；保留介于中性脂质与磷脂之间的中间杂质可被95%乙醇快速洗脱；磷脂峰集中在60~80 min，可被95%乙醇洗脱，各部分分离较好。之后逐渐提升载样量至5%和8%，以考察峰的展宽和交叉情况，图3b显示载样量增加至5%时，中性脂质与中间杂质的分离度迅速降低至勉强区分的程度，但中间杂质与磷脂的分离情况较好，有增加载样量的空间；图3c显示载样量增加至8%时，中间杂质与磷脂依然有一定的分离度，最终确定制备载样量为8%（进样体积240 mL）以保证分离效果和制备效率。

**图3:**
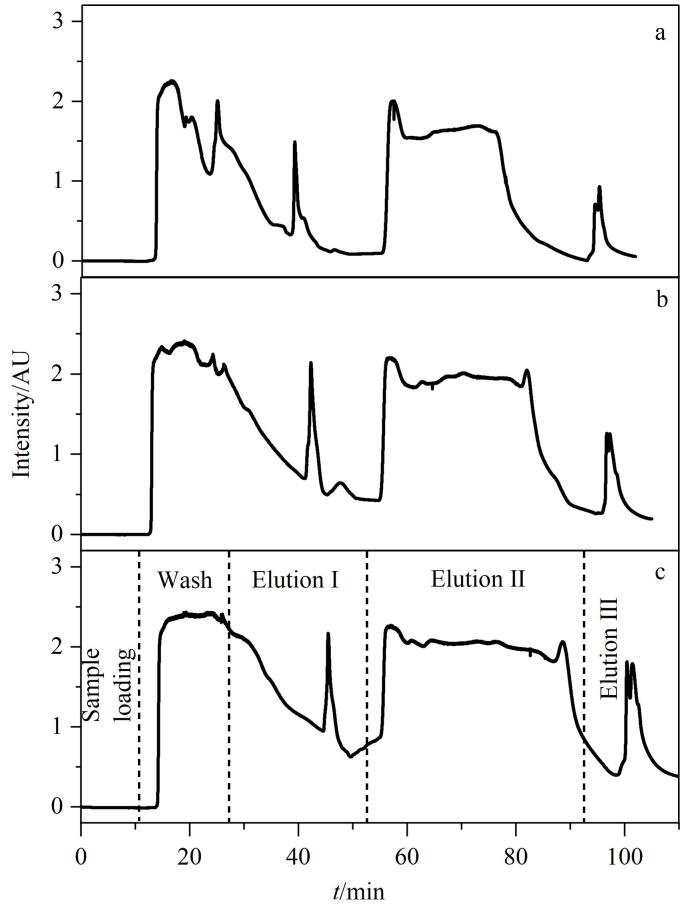
载样量对磷虾油制备的影响

### 2.3 亲水作用色谱制备效果评价

分别采用GB/T 5537-2008和GB 5009.168-2016测定4个馏分（Washing solution、Eluent Ⅰ、Eluent Ⅱ和Eluent Ⅲ）以及纯化前磷虾油中总磷脂以及EPA、DHA的含量。从表2数据可见，磷虾油中总磷脂含量为57.88%，经HILIC方法纯化后，Eluent Ⅱ中的磷脂含量提升至95.42%，该馏分的质量回收率为49.15%，总磷脂回收率为81%；EPA的含量略有提升，由15.70%提升至18.50%；DHA的含量与原样相近，非磷脂型EPA与DHA在Washing solution和Eluent Ⅰ馏分中被去除，导致磷脂富集馏分Eluent Ⅱ中EPA与DHA含量没有明显提升。

**表2:** 各样品中总磷脂、EPA、DHA的含量及质量回收率

Sample	Total phospholipid content/%	EPA content/%	DHA content/%	Mass recovery/%
Washing solution	2.54	8.52	5.42	44.16
Eluent Ⅰ	57.01	14.40	14.9	4.18
Eluent Ⅱ	95.42	18.50	7.18	49.15
Eluent Ⅲ	-	-	-	0.47
Krill oil	57.88	15.70	7.44	-

## 3 结论

本文通过优化流速、载样量、洗脱条件，确定了基于HILIC的南极磷虾油中磷脂的制备方法，得到总磷脂含量95.42%的产品，为后续高含量总磷脂和不同脂肪酸结合磷虾磷脂的开发及生产提供技术参考。
